# Level of Awareness of Dengue Disease among School Children in Gampaha District, Sri Lanka, and Effect of School-Based Health Education Programmes on Improving Knowledge and Practices

**DOI:** 10.1155/2019/3092073

**Published:** 2019-06-19

**Authors:** N. M. L. Radhika, Nayana Gunathilaka, Lahiru Udayanga, Anuradhani Kasturiratne, Wimaladharma Abeyewickreme

**Affiliations:** ^1^Central Environmental Authority, Battaramulla, Sri Lanka; ^2^Department of Parasitology, Faculty of Medicine, University of Kelaniya, Ragama, Sri Lanka; ^3^Department of Biosystems Engineering, Faculty of Agriculture and Plantation Management, Wayamba University, Makandura, Sri Lanka; ^4^Department of Public Health, Faculty of Medicine, University of Kelaniya, Ragama, Sri Lanka; ^5^Department of Paraclinical Sciences, Sir John Kotelawala Defence University, Ratmalana, Sri Lanka

## Abstract

**Introduction:**

Limited awareness and nonsystematized health education programmes have contributed adversely to the increase in dengue incidence at schools due to limited attention which has positively contributed to the increase in vector receptivity. The current study was conducted to evaluate the existing level of awareness of dengue infection among a selected group of school children and to assess the effectiveness of dengue awareness programmes to improve the existing knowledge and preventive practices on dengue.

**Methods:**

A cohort of 2,194 students (13–15 years old) from 10 schools at Kelaniya educational zone, Gampaha District, Western Province of Sri Lanka, was enrolled for the current study, which was conducted during 2015-2016. A self-administered questionnaire, comprising 20 questions, was used to evaluate the present knowledge of the students on various aspects of dengue. A structured awareness programme (2 hours) was conducted for students, followed by a reassessment. General Linear Model (GLM) and chi-square test of independence were used to investigate the variations in knowledge levels.

**Results:**

The majority of students were characterized by “Good” (46.31%, n= 1016) and “Moderate” (42.62%, n= 935) awareness, while only 2.92% (n= 64) of students fell into the “Excellent” (>80%) category prior to the awareness programme. Even though, existing knowledge of students about dengue ranged between “Moderate” and “Good” categories, awareness of “symptoms & patient care” and “control & prevention practices” were limited. After the programme, the awareness level reached the “Excellent” (41.84%, n=918) level indicating a significant increase by 38.92%, according to the chi-square test (p<0.05 at 95% level of confidence).

**Conclusion:**

We recommend implementing school-based educational programmes in order to raise the awareness and to translate knowledge into sound practice to control dengue disease epidemics in these areas.

## 1. Background

During past five decades, dengue fever (DF) has increased rapidly up to 30-fold, making it a global public health concern in the world [1–4]. It has been reported that 128 countries all over the world are endemic for DF, including Sri Lanka [5]. Dengue has reached the worst ever epidemic status in Sri Lanka recording the highest ever number of 186,101 cases in the year 2017. Ministry of Health recently declared 12 districts in the island as heavily affected areas. Among them over 50% of the total cases have been reported from the Western province of Sri Lanka, more specifically form Colombo and Gampaha districts indicating the first and second highest dengue cases, respectively [6].

Despite clinical progress towards the development of a vaccine against dengue virus (DENV), there are no proven vaccines available to be used in disease control [7]. Therefore, vector control efforts that aim to limit populations of the vector and prevent their invasion of other regions are considered as the only promising option at the moment [8, 9]. Hence, vector controlling authorities in Sri Lanka have vigilantly focused on source reduction and elimination of breeding habitats in the endemic regions [10].

The mosquito* Aedes aegypti* is the primary vector of DF, while* Aedes albopictus* remains as the secondary vector. Being hydrophilic mosquitoes, they prefer artificial containers for oviposition. Until recently, they were believed to oviposit in clear and clean water. However, some recent findings evidence that they can breed not only in clean and clear water bodies, but also in water with high organic matter content, low dissolved oxygen levels, and high salinity conditions [11].

Several studies have claimed that the majority of dengue infections occur peridomestically [12, 13], and therefore vector control has been embattled at residential areas. However, a recent study conducted in the city of Colombo, Sri Lanka, highlights the fact that, out of all identified breeding containers, about 11.9% residential premises, 66.7% schools, and 21.2% work or public sites had at least one container positive for larvae and or pupae [14]. The highest breeding rate has been observed at school premises.

Children spend a considerable amount of time in the school environment, which can facilitate the transmission of certain illnesses. As such, schools can serve as key locations for health promotion and disease prevention activities [15]. Health education and personal sanitation are necessary initial steps in modern control of mosquito borne diseases, as it involves removal of possible breeding sites of larvae [16]. For dengue control, public awareness and health education regarding the habitat and life cycle of the mosquito vector, as well as its physical control, are important in maintaining the vector population below risk levels. It is acceptable that there is severe lacking in the awareness as well as health education about dengue and its transmission among school communities [17]. Therefore, the objective of the present study was to determine the existing level of awareness of dengue infection and assess the effectiveness of dengue awareness programmes on school children to improve the existing knowledge and preventive practices on dengue. Hence, the study of this nature would be useful to reduce dengue incidence by public awareness and encouraged community participation.

## 2. Methods

### 2.1. Study Design

Cross-sectional study was conducted at selected ten schools in Kelaniya educational zone in Gampaha District, Western Province, during 2015-2016, where the dengue incidence is high. The study population was the secondary school students (13-15 years of age), who attended the day of the study and accepted to participate in it.

### 2.2. Sample Size Calculation

The sample size was calculated by using Epi Info programme formula. The sample size to achieve a precision of ±4 at a 95% confidence level was 172 from one school (172 X 10 = 1720 for all the schools), with a 90% likelihood of yielding a statistically significant result. During the fieldwork, the sample was increased to reach 2,194 students.

### 2.3. Data Collection

A standardized, confidential, anonymous, self-administered questionnaire was used in this study. A pilot study was conducted to assess the clarity of questions and the time required to complete the questionnaire. A reference number was assigned to each selected school and each student. The students were instructed to mention the reference number clearly in the questionnaire. The self-administered questionnaire comprised 20 multiple choice questions (MCQs) with 4 picks.

The questionnaire assessed the knowledge related to dengue infection, common symptoms, vector mosquitoes, breeding habitats of vectors, life cycle of the mosquito vector, common vector control measures/practices, distribution of dengue in the country, and the importance of solid waste management and 3 R's concept in controlling dengue incidence, under 4 main categories (disease transmission [No. of questions (m)= 4], vector ecology [m=4], symptoms & patient care [m=6], and control & prevention practices [m=6]).

An awareness programme covering the above aspects was conducted for a 2-hour duration including lectures using Microsoft PowerPoint, videos, and discussions. A new questionnaire with the same questions was given to each student after the awareness programme, and the awareness level was assessed as the post-evaluation. The students were guided to indicate the same reference number in the post-test questionnaire also.

Thorough entomological surveillance was conducted within the school premises on the same day and at intervals of one and two months after the awareness programme by using standard dipping, siphoning, and pipetting methods as recommended by the National Dengue Control Unit, Sri Lanka [18]. The larval surveillance activities were conducted after acquiring the informed written consent from the director of the educational zone and the principal of the relevant school. The collected specimens were identified up to the species level by trained entomological staff based on the standard morphological keys [19].

### 2.4. Data Analysis and Interpretation

The data from the pre- and post-questionnaires were coded and entered into IBM® SPSS® software version 23 (SPSS Inc., Chicago, IL). The responses to the knowledge questions were coded with one (1) for correct answers and zero (0) for incorrect and “do not know” answers, with a maximum of 20 points. The students' knowledge under 4 main categories was calculated prior to and after the awareness programme. Further, overall knowledge of students was calculated as a percentage (out of 20), and knowledge level was classified as Very Poor (<20%), Low (21–40%), Moderate (41–60%), Good (61–80%), and Excellent (81–100%). Based on the results of the entomological surveillance activities, the breeding site positivity was calculated as the percentage of* Aedes* larvae positive breeding sites over total breeding sites investigated.

### 2.5. Statistical Analysis

The significance of the variations in the knowledge of students on different aspects of dengue among the schools was investigated at both pre- and post-occasions by using General Linear Model (GLM). Chi-square test of independence was used to investigate the significance of different knowledge categories (based on the overall total). In addition, the effect of the school, school category (as defined by the Ministry of Education, Sri Lanka), and pre- and post-sampling occasion on the knowledge of students on dengue was statistically evaluated by using GLM followed by Tukey's pairwise comparison in SPSS (version 23).

The percentage of students belonging to different knowledge aspects was square-root transformed and the Distance-Based Redundancy Analysis (dbRDA) was performed to highlight and visually represent the underlying segregation patterns of the studied schools based on variations in the knowledge levels of students about the above-mentioned aspects of dengue using the Plymouth Routines in Multivariate Ecological Research version 6 (PRIMER 6). Further, the Analysis of Similarities (ANOSIM) was utilized to verify the overall clustering status of schools in terms of the knowledge of students about dengue.

### 2.6. Ethical Aspects

Ethical clearance for the study was obtained from the Ethics Review Committee (ERC) of the Faculty of Medicine, University of Kelaniya. Permission for the study was obtained from the Ministry of Education and the Provincial Director of Health Service, Western Province.

## 3. Results

### 3.1. Awareness among Students about Different Aspects of Dengue Disease

**Figure 1:**
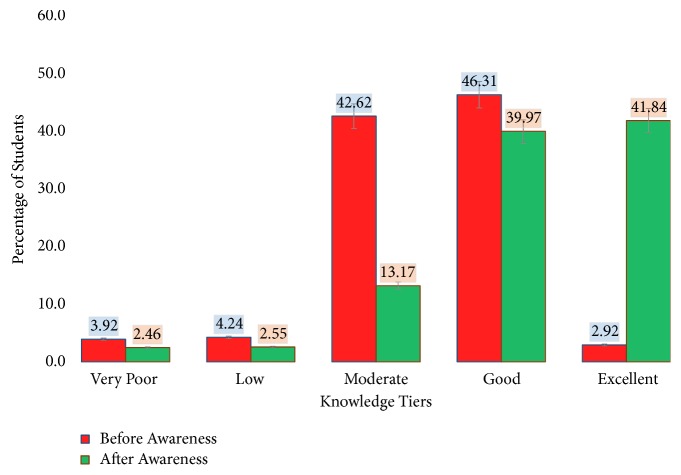
Cumulative knowledge of students about dengue prior to and after the awareness programme.

A total of 2,194 students were surveyed. Existing knowledge of the majority was characterized as “Good” (46.31%, n= 1016) followed by “Moderate” (42.62%, n= 935) levels. Only 2.92% (n= 64) of students were having an “Excellent” level of knowledge (>80%) on dengue, while 3.92% (n=86) of students were found to have a “Very Poor” level of awareness of dengue (Figure 1).

Overall, the mean knowledge score of students was 60.30±2.80 highlighting a “Moderate” to “Good” awareness level. It is important to indicate that the existing knowledge relevant to “symptoms & patient care” (2.76±0.04) and “control & prevention practices” for dengue (3.92±0.04) was highly limited prior to the awareness programme (Table 1). However, the awareness level of the above aspects reached “Excellent” level (41.84%, n=918), indicating a notable increase by 38.92%, after conducting the awareness programmes. Only 2.46% (n=54) of students fell into the “Very Poor” category.

Based on the statistics of the chi-square test of independence, knowledge levels in different categories differed significantly (p<0.05 at 95% level of confidence), before and after the awareness programme, whereby a significant increase of knowledge among the students on dengue could be recognized (Table 1).

### 3.2. Existing Knowledge of Students at Different Schools about Dengue

**Figure 2:**
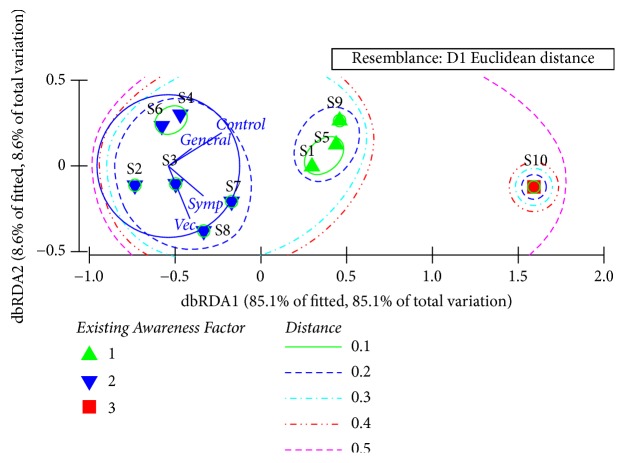
The dbRDA plot with clustering of schools based on the existing knowledge of students on different aspects of dengue.

As suggested by the dbRDA analysis, the studied sets of schools could be clustered into three categories at a Euclidean distance of 0.2, based on the overall existing knowledge of dengue. S10 that reported the highest mean level of knowledge (71.2%) remained alone, while S1, S5, and S9 created the second cluster with relatively higher overall knowledge levels. The other set of schools formulated the third cluster (Figure 2). The results of Analysis of Similarities (ANOSIM) confirmed the clustering pattern to be statistically significant with a global R value of 0.963 (p<0.05 at 95% level of confidence). As suggested by the dbRDA, knowledge of “controlling & prevention practices” and “symptoms & patient care” contributed immensely to the dbRDA 1 axis (with coefficients of 0.75 and 049, respectively).

On the other hand, awareness of “vector ecology” (0.73) and “symptoms & patient care” (0.47) significantly contributed to dbRDA 2 axis. Based on the dbRDA plot, existing knowledge of “vector ecology” and general knowledge and awareness of “symptoms & patient care” could be recognized as the core reasons for the clustering of S1, S5, and S9 together. Further, the spatial clustering status of S10 could be attributed to high knowledge in all the knowledge categories of dengue. On the other hand, the relatively lower performance of the students belonging to schools of S2, S3, S4, S6, S7, and S8 could be advocated as the reason for the emergence of them as a separate cluster. Statistics of the General Linear Model (GLM) also confirmed significant variations (p<0.05) in the mean scores of different knowledge categories and total scores among schools.

### 3.3. Variation in the Existing Knowledge after Conducting the Awareness Programme

It was noted that the percentage distribution of students into different knowledge categories differed significantly among schools (p<0.05 at 95% level of confidence). According to General Linear Model (GLM), only the “school” (p=0.001) influenced on the knowledge of the students, while “school category” (introduced by the Ministry of Education, Sri Lanka) remained nonsignificant (p=0.14) regarding dengue awareness (Table 2). The evaluation based awareness programmes (pre- and post-nature) [p=0.008], the interactive nature of such programmes, and school category (p=0.02) resulted in significant impacts (p<0.05 at 95% level of confidence) on raising the level of awareness.

**Figure 3:**
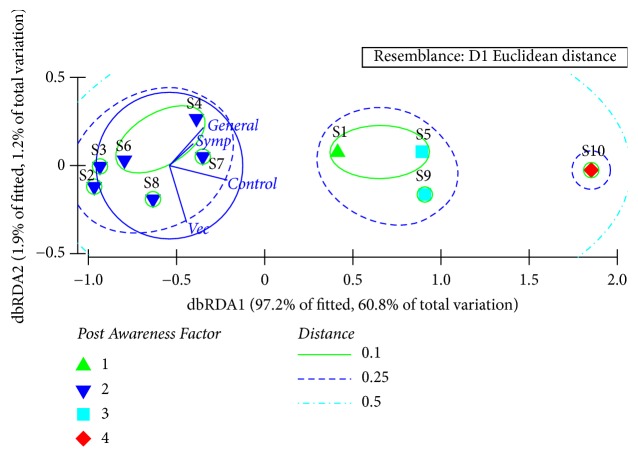
The dbRDA plot with clustering of schools based on the post-knowledge of dengue.

The dbRDA plot suggested the emergence of only 3 clusters, whereby S1, S5, and S9 were clustered together as a single cluster at a Euclidean distance of 0.25, while S10 remained independent. The remaining schools constituted the final cluster, may be due to low level of knowledge improvement. The exceptionally high performance in different awareness categories could be the reason for observing S10 as a separate cluster (Figure 3). With a global R value of 0.96 (p<0.05 at 95% level of confidence), the Analysis of Similarities (ANOSIM) further validated the above clustering pattern to be statistically significant.

### 3.4. Influence on the Vector Abundance

The results of the entomological surveillance activities conducted within the school premises are shown in Table 3. Among all the studied schools, discarded plastic containers (n=75) were the most abundant breeding site for* Aedes* larvae, followed by blocked roof gutters (n=48) and leaf axils (n=45), while the overall breeding site positivity for* Aedes* larvae remained as 32.13% before conducting the awareness programme. Interestingly, a significant reduction (p<0.05) of potential breeding sites and breeding sites positive for* Aedes* was observed at one-month and two-month intervals after the awareness programme. Therefore, it was evident that the awareness programme was effective in elevating the awareness and attitudes of the school communities in maintaining a safe environment with low degree of vector breeding.

## 4. Discussion

School-based education is an important compliment to community education, since transfer of knowledge and practice on a disease to children may result in disseminating the information to their households, which is crucial for source reduction at household level as a control measure for a mosquito borne diseases [7, 20]. Some studies conducted in other countries have shown that the primary school programmes have succeeded in increasing children's knowledge of dengue and participation in the prevention and control of dengue fever [9, 16, 21]. Hence, the present study evaluated the level of awareness of dengue under 4 major knowledge categories prior to and after a structured awareness programme.

In the present study, the study population had a good knowledge of “disease transmission”. The majority of students (over 75 %) knew that dengue is a viral disease transmitted by mosquito vectors belonging to genus* Aedes*. Several other studies conducted in Bangladesh, Puerto Rico, and Saudi Arabia have reported similar high rates of awareness (> 90%) about the mode of transmission of dengue among students [21–23]. Interestingly, 81.28% of the total student population were aware of the fact that dengue is a viral disease, while the majority of study population in several other studies have shown knowledge gaps regarding the viral transmission of dengue [23, 24].

In case of vector morphology, many students had a limited knowledge regarding the fact that the mosquito vectors can be identified as having distinctive black and white markings on the dorsal side of thorax, prior to the awareness programme. However, a previous study conducted in Saudi Arabia has reported moderate level of knowledge of vector morphology [25]. It is important to note that less than 30% of the students knew about high-risk areas for dengue in the country even though they were residing at a dengue high-risk area. Therefore, this may adversely affect the “disease transmission”, since the level of awareness and consciousness especially among community living in disease high-risk areas are interlinked with the implementation of preventive and control activities. A study covering the knowledge, attitudes, and practices of a dengue free individuals residing in Kandy has also documented a similar low level of knowledge about vector morphology that seems to be a key knowledge gap even among the general public [26].

Good knowledge of the signs and symptoms of DF is crucial to recognizing the disease and seeking appropriate healthcare [27]. Awareness about the disease in local community is one of the main factors that determine the success of a control programme [28]. Therefore, the knowledge of symptoms is essential to facilitate effective case management of dengue. Most students in the current study recognized fever as the prominent sign of the disease. However, only 8.24% (n=206) of the total population responded that the symptoms appear 4-6 days after infection. Overall, the study population indicated some gaps in knowledge of causes and disease recognition. Many studies from all over the world have reported similar findings, whereby notable limitations in knowledge of “symptoms & patient care” and management have been documented among students [23, 29, 30].

“Vector ecology” is another important aspect in eliminating the breeding habitats of* Aedes *mosquitoes, because knowledge is crucial to identifying breeding sites and initiating control approaches [17]. The current study population had considerably a good awareness level concerning “vector ecology”, which is a positive factor for identification of breeding sites and thereby reduction of breeding habitats. Most children responded that the proper waste disposal and management are ideal in dengue prevention by reducing the receptivity of disease vectors. Some studies have also emphasized proper waste disposal and solid waste management as key infrastructural features for control of dengue transmission [17, 26, 31]. Unlike previous studies conducted in Bangkok, the present study population has notably a higher level of knowledge of the “control & prevention practices” for dengue, which are crucial factors in community-based management of dengue [32].

A previous study conducted in Sri Lanka has highlighted the fact that the community at schools and public work places did not voluntarily implement vector control measures [14]. Therefore, schools and public sites have been identified as the highest risk areas for occurrence of productive breeding sites, thereby causing serious shortcomings in preventive measures. The study has also urged the need for educating the school communities regarding the disease. It was found that twinning of social participation and environmental management improves the effectiveness of conventional dengue control programmes and significantly reduces vector densities [33]. Hence, proactive health education through appropriate awareness programmes can strengthen and encourage community participation for dengue epidemic management [34].

The most severe dengue epidemic of Sri Lanka occurred in 2017, reporting 186,101 suspected dengue cases from the country, where the district of Gampaha remained as the second most high-risk area for dengue accounting for 15.66 % (n= 29, 150). Sudden shift of dengue viral serotype from DENV-1 and DENV-4 to DENV-2 [6, 35], the overwhelmed situation of the country due to heavy rains and flooding events that resulted in a higher abundance of potential breeding sites, failures in clearing rain-soaked garbage, and poor waste management and disposal practices could be attributed to the higher number of dengue cases reported in the country [36]. However, the severity of this condition could have been contained if proper preventive practices were followed by the local communities. Therefore, this emphasizes that mere increase in knowledge in prevention and control is not effective in dengue control, while attitudes of the local communities should be developed to ensure translation of knowledge into practices [17, 37, 38].

In general, the awareness levels about dengue varies from one school to another even though all these are located within the same educational zone in the same district. This may be due to some changes in the educational level of students, availability of resources, dedication of staff members, existing awareness programmes, and dengue prevention activities at school level. Therefore, a common awareness activity covering whole aspects should be conducted among all schools along with organized dengue control activities at schools. The content of the message and mode or frequency of delivery remain as the most difficult aspects in health education development. Hence, messages need to be relevant to people's daily practices and offer practical and effective activities. Therefore, it is recommended that school-based educational programmes and social mobilizations should be implemented in order to raise the awareness, to translate knowledge into sound practice within all schools in disease endemic areas, and to control dengue disease epidemics.

## 5. Conclusion

Initial knowledge of students about “symptoms & patient care” and “control & prevention practices” for dengue were highly limited prior to the awareness programme. However, awareness level was increased significantly after the awareness programme. The present study concludes that the school-based health education and awareness programmes are valuable to uplift the level of awareness of diseases like dengue. Therefore, it is timely to recommend school-based educational programmes and social mobilizations in order to encourage community participation for dengue control.

## Figures and Tables

**Table 1:** Average mean scores for different knowledge categories and overall knowledge of students on dengue prior and after awareness programme.

Knowledge category	Prior	After	p value
Disease transmission	2.58 ± 0.023	3.28 ± 0.026	0.02
Vector ecology	2.97 ± 0.027	3.40 ± 0.027	
Symptoms and patient care	2.76 ± 0.036	4.04 ± 0.045	
Control and prevention practices	3.92 ± 0.043	4.45 ± 0.043	
*Overall knowledge score (out of 100%)*	*60.30 ± 2.80*	*77.89 ± 23.25*	

**Table 2:** Average mean scores for different knowledge categories and overall knowledge of students on dengue.

School reference number	Average Total Score (%)		p value for the GLM
	Pre	Post	
S 1^f^	59.65	81.40	For variance among schools = 0.001
S 2^b,c^	55.65	77.20	
S 3^b^	58.95	68.70	
S 4^b^	55.80	73.80	
S 5^e^	63.45	85.75	
S 6^a^	54.05	69.30	For variance among pre & post = 0.008
S 7^d^	60.00	76.75	
S 8^b^	57.90	68.50	
S 9^f^	67.95	87.25	
S 10^d^	71.20	90.25	

**Table 3:** Summarized findings of the entomological surveillance activities

Breeding site	Before intervention		One month after intervention		Two months after intervention	
	Number of potential breeding sites	Number of positive breeding sites	Number of Potential breeding sites	Number of positive breeding sites	Number of potential breeding sites	Number of positive breeding sites
Leaf axils	45	14	24	5	13	2
Tree hole	25	8	13	3	1	0
Coconut shell	31	7	8	2	0	0
Discarded plastic containers	75	24	8	1	8	1
Discarded metal containers	19	9	11	3	2	0
Discarded glass containers	12	7	7	2	4	1
Flower pots	22	4	19	2	15	2
Discarded tire	16	5	8	1	0	0
Blocked drains	12	2	5	1	4	1
Blocked roof gutters	48	16	28	8	3	0
Total habitats	305	98	131	28	50	7
Breeding Site Positivity for *Aedes *larvae (%)	32.13		21.37		14.0	

## Data Availability

The data used to support the findings of this study are included within the article.

## Abbreviations

ANOSIM: Analysis of Similarities
dbRDA: Distance-Based Redundancy Analysis
DF: Dengue fever
GLM: General Linear Model
PRIMER: Plymouth Routines in Multivariate Ecological Research.
